# Reproductive Performance and Partial Budget Analysis of a Prostaglandin or a Modified Ovsynch Protocol in Autumn Calving Dairy Herds

**DOI:** 10.3390/ani11041031

**Published:** 2021-04-06

**Authors:** George Lindley, Jim Willshire, Steven Van Winden

**Affiliations:** 1 Royal Veterinary College, Hawkshead Lane, North Mymms, Hatfield, Hertfordshire AL9 7TA, UK; svwinden@rvc.ac.uk; 2 Endell Farm Vets, 49 Endless Street, Salisbury SP1 3UH, UK; jim@endellfarmvets.co.uk

**Keywords:** dairy, cattle, reproduction, prostaglandin, Ovsynch, progesterone, partial budget

## Abstract

**Simple Summary:**

In autumn calving dairy herds cattle must conceive within a restricted timeframe to maintain the seasonality of calving. Cattle not observed in estrus prior to the mating start date are frequently treated with a single prostaglandin injection even though its relative effectiveness is unknown. The perceived cost of other treatments, such as a modified Ovsynch protocol, may be a reason why they are less favored. This study compares the reproductive and economic outcomes associated with treating cows not observed in estrus prior to breeding with a modified Ovsynch protocol, in comparison to a single prostaglandin injection. The main inputs influencing profitability are identified, and differences in dry-off strategy are considered. From the analyses, cows treated with a modified Ovsynch protocol had a greater likelihood of conception at the beginning of, and throughout, the breeding season. Differences in barren rate and the amount of milk produced were the main variables affecting profitability, the latter strongly influenced by drying-off policy. Farmers and advisors should consider the relative performance of their breeding strategies as well as their own management policies to optimize reproductive and economic performance.

**Abstract:**

In autumn calving dairy herds, treatment of cattle not observed in estrus prior to the breeding season is common. Routinely, a single prostaglandin or a modified Ovsynch (MOFT) protocol are used—without evidence of their relative effectiveness. This study compares the effects on conception, associated timing, and profitability of administering cows with prostaglandin or MOFT treatment. A hundred and ninety-two Holstein-Friesian cows from three herds without an observed estrus within 28-days before mating start date were randomly treated with d-cloprostenol (PGOD) or an 8-day MOFT protocol. The association of treatment and calving-breeding start-date interval (CBSI) on the risk of conception were investigated. Partial budget, sensitivity analysis, and Monte Carlo simulation was used to assess economic performance, identify critical input variables, and explore the effects of input uncertainties on model output. There was a significant association between MOFT treatment and conception during 21 and 84 days after mating start date, compared to PGOD. MOFT treatment was associated with a mean net benefit of £58.21 (sd £19.42) and £27.29 (sd £17.75) per cow for herds with a fixed or variable dry-off date, respectively. The relative profitability of an MOFT protocol is dependent on its effects on barren rate and herd dry-off strategy.

## 1. Introduction

Seasonal calving dairy systems aim to achieve a compact calving period, allowing calving, breeding, lactation, and dry-off to be coordinated for the entire herd within a discrete temporal pattern. Within the United Kingdom, autumn calving dairy herds seek to maximize milk production from grazed or harvested forage; during peak lactation cows are kept indoors, allowing nutrition to be optimized, whilst during spring and summer cows graze low-cost, high quality pasture, which may improve lactation persistency [1]. In order to maintain their inherent seasonality, the commencement of breeding is determined by the desired start of calving during the subsequent year and cows must conceive within a restricted timeframe [2]. Associations between timely conception and financial performance have been identified [3,4] and reproductive efficiency is commonly described using interval-based measures such as 21, 42, and 84 day in-calf rate [5]. Improvements require submission and conception rates to be maximized, particularly at the beginning of breeding [6]. For this reason, estrus detection is usually performed prior to the mating start date [7,8], allowing cows without an observed estrus to be swiftly identified and treated.

The causes of unidentified estrus may be broadly classified by means of the physiologic and pathologic follicular or luteal dynamics that occur [9,10,11]. Subestrus describes individuals that are ovulating but are not identified (“not bulling”). This ovulation without behavioral expression, or “silent heat”, may occur in up to half of lactating dairy cattle [12,13]. Alternatively, estrus behavior is not detected as a result of herdsperson, management, or environmental factors [14]. Subestrus cattle do ovulate and the presence of a corpus luteum, identified using, transrectal ultrasonography or progesterone assays, allows confirmation [15,16].

Unidentified estrus may also be the result of anestrus, characterized by the absence of progesterone production or a corpus luteum [9]. It is caused by follicular growth which does not progress beyond emergence or deviation, or due to cystic ovarian follicular disease [17,18]. Whilst a period of anestrus may be expected postpartum [19], extended postpartum anestrus intervals may occur in up to 20% of the breeding herd and are associated with increased calving to conception intervals, as well as an increased risk of culling [20,21]. Prompt treatment and resolution of anovulatory disorders is necessary if reproductive performance during breeding is to be optimized.

One common management strategy for cows not identified in estrus involves treatment with a prostaglandin-based therapeutic without previous ovarian examination [22,23]. Biologically, luteolysis will occur in individuals in diestrus [24] and responsive cows will return to estrus between two and seven days after treatment [25,26]. If timed strategically, this may allow cows who have unidentified estrus to be inseminated within the first week of breeding. However, this approach is ineffective in up to half of treated cows, the result of an absent or unresponsive corpus luteum or an unidentified estrus event following treatment [15]. In comparison Ovsynch-based protocols can achieve synchronization of large groups of cattle, may be used as a treatment for anovulatory anestrus [27,28] and can be used alongside fixed-time artificial insemination, eliminating the requirement for estrus detection [29]. However, to be optimized they require multiple injections administered at specific times, incurring a greater direct cost of initial treatment. Within the United Kingdom, no economic analysis of the relative profitability of a modified Ovsynch protocol has been performed in autumn-calving herds.

The aims of this study are to (i) compare the effect of a single prostaglandin treatment with a modified Ovsynch protocol administered without ovarian examination to cows not observed in estrus during 28-days before the mating start date on the timing and extent of conception during an 84-day breeding season in autumn calving dairy herds in the UK; (ii) compare the relative economic net benefit of a modified Ovsynch protocol in comparison to a prostaglandin protocol administered without ovarian examination to cows without an observed estrus during 28-days before treatment, at mating start date; (iii) explore the effects of uncertainties in input variables and differences in lactational dry-off management strategy on output, measured as the net benefit per cow treated in pounds sterling.

## 2. Materials and Methods

### 2.1. Ethics Statement

This study was undertaken with prior approval of the Royal Veterinary College’s Clinical Research Ethical Review Board (ref: URN 2019 1921-3) and was granted an Animal Test Certificate by the Veterinary Medicines Directorate (ref: ATC-S-135).

### 2.2. Reference Population

A prospective cohort study was performed on three commercial dairy herds in South West England, details of which are summarized in Table 1. In each herd cows were milked twice daily. Cows grazed grass leys during summer and were moved into housing based upon climatic conditions and pasture availability. In all herds, cows were housed prior to the onset of heat detection. Sample size estimations were produced from a priori power calculations. Based upon a Type 1 error of 0.05, a power of 0.8, a follow up period of 84 days and an estimated hazard ratio of 1.5, 96 animals would be required per treatment group. Subsequently, a convenience sample of 192 predominantly Holstein-Friesian cattle were enrolled during October 2019. Selected herds operated seasonal autumn calving systems with 90% of cows calving between August and October 2019. For each herd, mating start date was set prospectively. All herds performed estrus detection using tail paint for a duration of 28 days prior to enrolment. Cows not detected in estrus by the time of enrolment at mating start date (day 0) were recruited for the study. Nulliparous heifers, cows with a calving interval >365 days, cattle that were systemically unwell or with abnormal vaginal discharge at enrolment were excluded from the study. Herds 2 and 3 bred cows to artificial insemination throughout the 84-day study period, whilst herd 1 performed artificial insemination for the first 42 days, followed by natural service.

### 2.3. Study Design

**Figure 1:**
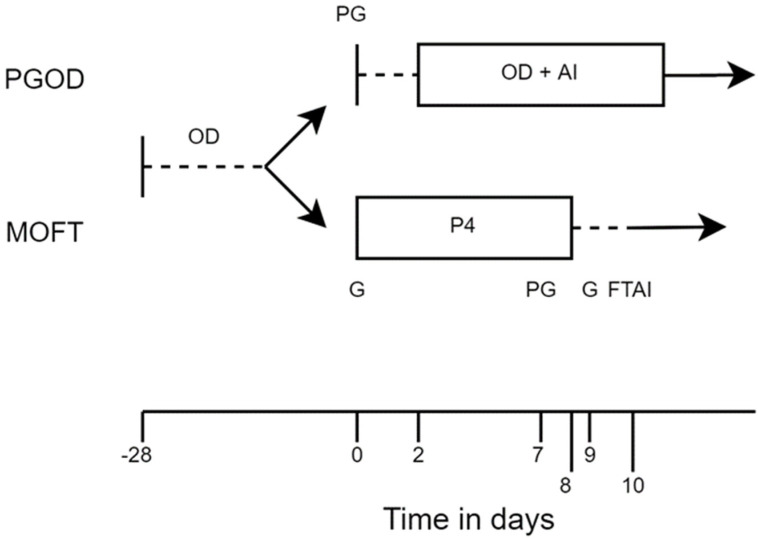
Experimental design of a randomized trial to compare the effect of two different treatment protocols (i) PGOD: 0.15 mg d-cloprostenol sodium (PG) intramuscularly at day 0 and insemination (AI) following observed estrus (OD) from day 2 onwards; (ii) MOFT: 100 μg of gonadorelin acetate (G) intramuscularly and an intravaginal device containing 1.38 g progesterone (P4) at day 0, 0.15 mg d-cloprostenol sodium intramuscularly at day 7, removal of the intravaginal device at day 8 and 100 μg of gonadorelin acetate intramuscularly at day 9, 24 h after device removal. Fixed time artificial insemination (FTAI) took place 54 ± 6 h after device removal (10.25 ± 0.25 days); administered to cows not observed in estrus for 28 days prior mating start date at mating start date (day 0).

Cows were enrolled into the study on mating start date, day 0. All recruited cows were allocated to a treatment group based on freeze-brand number (Figure 1). Cows with an even freeze-brand number were treated with 0.15 mg d-cloprostenol sodium (Prellim; Zoetis, Surrey, UK) intramuscularly at day 0 and were eligible for insemination following observed estrus from day 2 onwards (PGOD). Cows with an odd freeze-brand number were administered 100 μg of gonadorelin acetate (Acegon; Zoetis, Surrey, UK) intramuscularly and an intravaginal device containing 1.38 g progesterone (CIDR; Zoetis, Surrey, UK) at day 0, 0.15 mg d-cloprostenol sodium (Prellim; Zoetis, Surrey, UK) intramuscularly at day 7, removal of the intravaginal device (CIDR; Zoetis, Surrey, UK) at day 8 and 100 μg of gonadorelin acetate (Acegon; Zoetis, Surrey, UK) intramuscularly at day 9. Fixed time artificial insemination took place between day 10 and day 10.5. Cattle showing signs of estrus after removal of the intravaginal device (CIDR; Zoetis, Surrey, UK) were not given the final injection of 100 μg of gonadorelin acetate (Acegon; Zoetis, Surrey, UK) and were inseminated according to the a.m.:p.m. rule, [12,30] or at the time of fixed time artificial insemination, whichever was sooner (MOFT).

### 2.4. Analytical Methods

At enrolment (day 0) cows were body condition scored (BCS) on a scale of 1 to 5 (at 0.25 intervals) as described by Edmondson et al. [31]. For each herd, background and reproduction data including calving date, parity, age (in months), service dates and results of pregnancy diagnoses were recorded on a database (UNIFORM-Agri; Somerset, UK; Farmwizard; Peterborough, UK) and data was extracted following the end of breeding. Data were collated onto a spreadsheet (Excel for Office 365; Microsoft, WA, USA) and the interval between calving and the start of breeding was calculated for each cow (CBSI).

### 2.5. Statistical Methods

Data were imported into SPSS V26.0 (IBM Corp; Armonk, NY, USA) for statistical analysis. Frequency distributions of continuous data were produced; and assessment of kurtosis and skewness was used to determine the normality of each distribution. Summary statistics of categorical data were produced. For all analyses, treatment was defined as the primary exposure variable. The recorded factors and covariates; farm, CBSI, treatment, parity, age; and BCS were also considered for inclusion in the analysis. Parity was classified into 3 groups; 1st, 2nd, and ≥3rd. Body condition score was classified into two groups; ≤2.5 and ≥2.75. Breeding-conception interval was categorized into categorical variables based upon the timing of conception for logistic regression analysis and retained as a continuous variable for survival analysis. All analyses were performed on an intention-to-treat basis.

Separate logistic regression models were used to determine the effect of treatment on the binary outcome of conception within 21 and 42 days after day 0. A stepwise approach was taken with the variable “farm” initially forced into the model. Variables were retained in the model based upon the likelihood ratio test and a significant associated Chi-squared statistic (*p* < 0.05). Model checking revealed that the assumption of linearity of the logit had been breached and resultantly CBSI was transformed into a categorical predictor with three groups: <60 days, 60–90 days, and >90 days. Multicollinearity among the independent variables was not identified, nor were any significant two-way interactions between any factors or covariates. Model fit was assessed by visual examination of residual plots [32]. The influence of residuals were evaluated using DFBeta, leverage, and Cook’s distance statistics.

Kaplan–Meier analysis, stratified by treatment, was used to identify median conception time and produce time point estimates. Cumulative pregnancy curves were produced to assess temporal patterns between treatment groups. The breeding period was defined as 84 days and all cows that had not conceived or were not inseminated by this point were right censored. Log-rank testing was used to determine if a significant difference in survival distributions was present between treatment groups. Due to differences in the timing of first insemination between treatment groups, the effects of treatment varied over time. In order to satisfy the proportional hazards assumption during subsequent cox regression analysis, mating start date was defined separately for each treatment group as the date of first insemination for all survival analyses.

Cox regression analysis was used to investigate the association between treatment and the hazard of conception. Model building was performed as for the logistic regression analysis. The assumption of proportional hazards was not breached when evaluated by adding the interaction between treatment and time to the model. Influential observations were assessed using DFBeta measurements and the model was refitted without influential observations to assess differences in associated Chi-square values. Hazard ratios were produced to describe the relative risk of conception based on comparison of event rates in the exposure variable.

### 2.6. Partial Budget Analysis

A Partial budget model was created using a spreadsheet (Excel for Office 365; Microsoft, WA, USA). The additional income, costs saved, revenue foregone and additional costs of using MOFT in comparison to PGOD was calculated. Analysis was performed for herds with a fixed, or variable dry-off date. All input values for the partial budget analysis and sensitivity analysis are provided within Supplementary Materials (Table S1).

#### 2.6.1. Additional Income

For herds where dry-off was performed on fixed calendar date, additional milk produced during the subsequent lactation was calculated (per cow). Additional days lactating was calculated as the difference in mean calving conception interval between treatment groups from the study, 4 days. Daily milk yield was calculated from mean 305-day milk yield between farms. The value of extra milk produced was estimated from the average price paid by all dairies within Great Britain during September 2020 [33]. For the analysis of herds where dry-off was not a fixed date, this calculation was omitted.

Income from additional calves produced (a consequence of a reduced barren rate) was calculated per 100 cows calving before additional income per cow calving was determined. Using insemination records and transrectal ultrasonography results, pregnancy diagnoses data was computed. The proportion of cows conceiving within the first or second 42 days after mating start date was calculated for both treatment groups using time-point estimates produced from Kaplan–Meier analysis. Cows conceiving within the first half of the breeding season were assumed to have conceived to conventional Holstein semen, whereas those conceiving within the second half of the breeding season were assumed to have conceived to conventional Hereford semen. Calf values were estimated for male and female Holstein-Friesian or Hereford cross calves under three weeks of age from average sales prices of auction markets in Great Britain [34]. The ratio of male:female calves born was estimated as 0.52:0.48 [35].

#### 2.6.2. Reduced Costs

From the study, it was calculated that conception rate to first insemination between treatment groups was 5% greater in the MOFT group than the PGOD group. Conception rate to subsequent inseminations was calculated overall for each treatment group and the mean inseminations required per conception for each treatment group estimated as 1.72 and 1.65 inseminations per pregnancy per cow for PGOD and MOFT, respectively. This data was combined with an estimation of cost per insemination to produce values for the overall cost of inseminations per cow per treatment group.

Survival analysis data was used to calculate the proportion of cows failing to conceive during the breeding season for each treatment group. Cows that did not conceive during breeding were classified as barren and were assumed to be culled. Cull cow value was estimated from mean auction market prices in Great Britain during October 2020 based upon a liveweight of 650 kg per cow [36]. This value was subtracted from the associated cost of rearing a replacement heifer in an autumn calving system from birth until first calving [37] to represent the reduced costs associated with a reduction in barren cows at the end of breeding. During the sensitivity analysis, when differences in barren rate were set as 0%, these costs were omitted.

#### 2.6.3. Returns Foregone

No foregone returns were considered.

#### 2.6.4. Extra Costs

The additional costs of treatment were calculated by subtracting the cost of the PGOD protocol from the cost of the MOFT protocol. An additional feed cost was also considered; a consequence of cows calving earlier during the subsequent calving season and requiring a lactational ration for a longer period. The estimated costs of feed per liter of milk produced [38] were multiplied with mean daily milk production and the mean reduction in calving-conception interval to produce this figure.

### 2.7. Sensitivity Analysis

Sensitivity analysis allows identification of input variables with the greatest influence on profitability [39]. Analysis was performed using SensIT^®^ (San Francisco, CA, USA). Values for low, expected and high ranges were derived from mean values and their associated 95% confidence intervals from the study or based upon estimates of variation in the expected value. Key input variables and their values are displayed in Table 2. Analysis was performed for four scenarios: (a) herds with a fixed dry-off date and a reduced barren rate; (b) herds with a fixed dry-off date but no improvement in barren rate; (c) herds with a variable dry-off date and a reduced barren rate; and (d) herds with a variable dry-off but no improvement in barren rate.

### 2.8. Monte Carlo Simulation

Monte Carlo uncertainty assessment specifies a probability distribution for each sensitivity parameter and repeats the sensitivity analysis multiple times, allowing the probability distribution of the output to be appropriately described [40]. Monte Carlo simulation of net benefit was developed using SimVoi^®^ (San Francisco, CA, USA). Input values were produced using the associated semi-random number generator function, "RandTriangular", which allows data to be selected from a triangular probability density function taking into account the minimum, maximum, and “most likely” values based upon those presented within the sensitivity analysis. The analysis was performed for 10,000 iterations for each of the same four scenarios as described for the sensitivity analysis.

## 3. Results

### 3.1. Descriptive Analysis

One hundred and ninety cows were included in the final analysis on an intention-to-treat basis. Ninety cows were enrolled into the PGOD protocol and one hundred cows were enrolled into the MOFT protocol. Two cows from the PGOD group were not included in the analysis; a consequence of incorrect freeze brand number recording and culling prior to pregnancy diagnosis, respectively. In all herds, pregnancy diagnoses of inseminations performed until 84 days after the mating start date were followed up and included in the analysis. Conception rate to first insemination was 52% (n = 46) and 57% (n = 57) within the PGOD and MOFT groups, respectively. Twenty-one day submission rate and 21 day in-calf rate was 86% (n = 76) and 44% (n = 40) for PGOD treatment, and 96% (n = 96) and 56% (n = 56) for MOFT treatment. Forty-two day in-calf rate was 70% (n = 63) and 72% (n = 72), whilst 84 day in-calf rate was 77% (n = 70) and 85% (n = 85) for PGOD and MOFT treatment, respectively. Mean calving-first service interval and mean calving conception interval was 80 (SD 27) and 95 (SD 29) days for PGOD treatment, and 79 (SD 22) and 91 (SD 22) days for MOFT treatment. Table 3 summarizes the key reproductive parameters of the study population.

### 3.2. Logistic Regression Analysis

Conception within 21 days was significantly associated with treatment, farm, and CBSI, whereas conception within 42 days was significantly associated with farm and CBSI only. When compared to PGOD treatment, cows in the MOFT group had a greater probability of conception within 21 (OR; 2.08, 95% CI 1.11–3.91, *p* = 0.02) but not 42 (OR 1.15, 95% CI 0.58–2.29, *p* = 0.69) days. An increased CBSI was associated with an improved probability of conception in both analyses. When compared to a reference CBSI of 0–59 days, a CBSI of 60–90 days was associated with a tendency for increased odds of conception within 21 (OR 1.95, 95% CI 1.36–9.59, *p* = 0.07) days and a significantly increased odds of conception within 42 (OR 2.17, 95% CI 1.01–4.57, *p* = 0.04) days, whereas a CBSI of >90 days was associated with a significantly greater risk of conception within both 21 (OR 3.61, 95% CI 1.36–9.59, *p* = 0.01) and 42 (OR 4.97, 95% CI 1.55–15.90, *p* < 0.01) days.

### 3.3. Survival Analysis

#### 3.3.1. Kaplan–Meier Analysis

**Figure 2:**
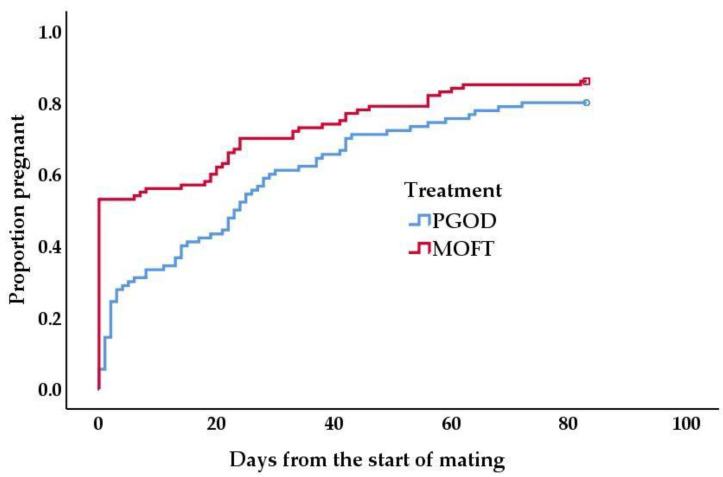
Kaplan–Meier analysis of cumulative pregnancy (1—survival) for: (i) red line, MOFT; (ii) blue line, PGOD. Log-rank testing identified a significant difference in survival distributions between treatment groups (*p* = 0.03). Survival analysis was recorded for a duration of 84 days and cows that had not conceived or had not been inseminated by this time were right censored as displayed by a: (i) red square, MOFT; (ii) blue circle, PGOD.

A significant difference between survival distribution of each treatment group was identified (Log-rank, *p* = 0.03). Median estimated conception time was 0 days and 23 (95% CI 18–28) days in the MOFT and PGOD groups, respectively. Time point estimates of cumulative pregnancy (1—survival) were produced for the MOFT (21 d, 0.63, SE 0.05; 42 d, 0.77, SE 0.04; 84 d, 0.86, SE 0.04) and PGOD (21 d, 0.44, SE 0.05; 42 d, 0.70, SE 0.05; 84 d, 0.80, SE 0.04) groups. At the end of the follow up period, 18 cows and 14 cows were right censored in the PGOD and MOFT groups, respectively. Kaplan-Meier plots for each treatment group are shown in Figure 2. 

#### 3.3.2. Cox Regression Analysis

Treatment, farm and CBSI were found to be predictive of conception in the model. After adjusting for the other covariates and when compared to the PGOD group, MOFT treatment was associated with an increased hazard of conception (HR 1.40, *p* = 0.04). A small but significant CBSI effect was also found; each day increase in CBSI was associated with a 1% increase in the hazard of pregnancy (HR 1.01, *p* = 0.01). When assessed in comparison to Farm 1, considerable variation in the risk of conception between farms was detected (Farm 2, HR 1.9, *p* = 0.05; Farm 3, HR 0.75, *p* = 0.11).

### 3.4. Cost–Benefit Analysis

Partial budget analysis calculated a net benefit of £53.42 and £26.54 per cow treated with the MOFT protocol for farms with a fixed dry-off date and variable dry-off date, respectively.

#### 3.4.1. Sensitivity Analysis

**Figure 3:**
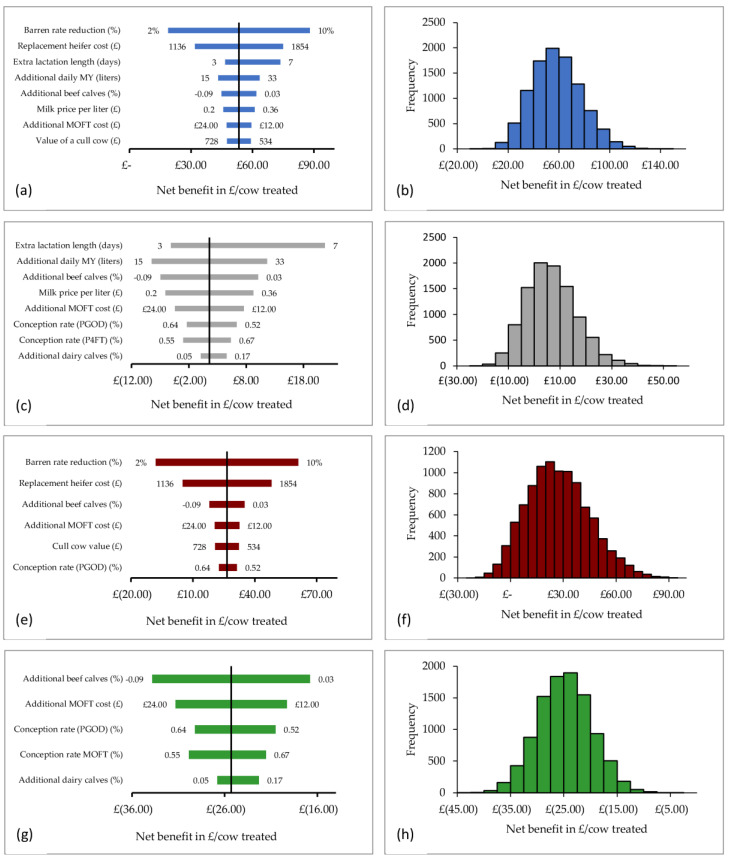
Sensitivity analysis and Monte Carlo simulation showing the influence of input variable uncertainties on the net benefit value in pounds sterling per cow treated; (**a**,**b**) herds with a fixed dry-off date and a reduced barren rate; (**c**,**d**) herds with a fixed dry-off date but no improvement in barren rate; (**e**,**f**) herds with a variable dry-off date and a reduced barren rate; (**g**,**h**) herds with a variable dry-off but no improvement in barren rate. X-axis values within brackets represent negative values.

As displayed in Figure 3, in circumstances where MOFT treatment resulted in an improvement in barren rate at the end of breeding, percentage reduction in barren rate and the cost of a replacement heifer were the input variables with the greatest influence on net return, regardless of herd dry-off policy. They accounted for 54.6% and 21.2%, 65.8% and 25.2% of the total variation in output in herds with a fixed and variable dry-off dates, respectively.

In comparison, in herds with a fixed dry-off date when MOFT treatment did not reduce barren rate at the end of breeding, the input variables with the greatest influence on net return were increased lactation length and additional milk produced per day, accounting for 36.6% and 20.6% of the total variation in output, respectively. When barren rate differences between treatment groups were set to 0% in herds with a variable dry-off date, the proportion of beef calves produced during the breeding season and the additional cost of treatment with a progesterone protocol had the greatest influence on net return, accounting for 47.6% and 23.7% of the total variation in output.

#### 3.4.2. Monte Carlo Simulation

Results of the Monte Carlo simulation are described below and summarized in Figure 3:

1. Herds with a fixed dry-off date and a reduced barren rate: mean net return was £58.21 (SD £19.42; first quartile £44.37; third quartile £71.09) per cow treated with the MOFT protocol.
2. Herds with a fixed dry-off date but no improvement in barren rate: mean net return was £6.62 (SD £9.76; first quartile £−0.35; third quartile £12.73) per cow treated with the MOFT protocol.
3. Herds with a variable dry-off date and a reduced barren rate: mean net return was £27.29 (SD £17.75; first quartile £14.50; third quartile £38.87) per cow treated with the MOFT protocol.
4. Herds with a variable dry-off but no improvement in barren rate: mean net return was £−24.83 (SD £5.03; first quartile £−28.28; third quartile −21.42) per cow treated with the MOFT protocol.

## 4. Discussion

This study compared the effects of treatment with a synthetic prostaglandin analogue versus a modified Ovsynch protocol, administered at mating start date to cows not identified in estrus prior to breeding in autumn calving dairy herds in the United Kingdom. When overall treatment effects were compared throughout the breeding period, cows in the MOFT group were 1.4 times more likely to conceive (HR 1.40, *p* = 0.04) than cows in the PGOD group during an 84-day breeding season. A significant association between MOFT treatment and conception within the first 21 (OR 2.08, *p* = 0.02) days breeding was also found. Subsequent partial budget analysis investigated associations between MOFT treatment and profitability, when compared to PGOD treatment. The relative profitability of MOFT treatment was substantially influenced by its effects on reducing barren rate by the end of breeding. In circumstances where MOFT treatment was associated with improvements in barren rate, Monte Carlo simulation predicted a mean net benefit of £58.21 (SD £19.42; first quartile £44.37; third quartile £71.09) and £27.29 (SD £17.75; first quartile £14.50; third quartile £38.87) per cow treated for herds with a fixed and variable dry-off policy, respectively. The relative profitability of MOFT treatment in circumstances where barren rate was not improved were less clear. Mean net benefit was £6.62 (SD £9.76; first quartile £−0.35; third quartile £12.73) and £−24.83 (SD £5.03; first quartile £−28.28; third quartile −21.42) per cow treated for herds with a fixed and variable dry-off policy, respectively.

The results of this study are in agreement with those of Mcdougall [4] and Beukes [2], who found that in herds in New Zealand with no difference in barren rate and a fixed dry-off date, the additional milk produced as a result of an extended subsequent lactation was the main factor influencing the profitability of treatment of cows without an observed estrus. Nonetheless, this is the first study evaluating the cost–benefit of a modified Ovsynch in herds with variable dry off dates, a common management practice for autumn calving dairy herds within the United Kingdom. When included in the economic analysis, the extent to which MOFT reduced barren rate, in comparison to PGOD treatment, was the main predictor of profitability regardless of dry-off policy. Nonetheless, equivocal outcomes between studies demonstrate that inter-farm variability in the effectiveness of MOFT treatment is common [41,42]. As a consequence, and in order to account for differences in the effectiveness of MOFT treatment between farms, the analysis performed in this study assessed situations with and without differences in barren rate between treatments, allowing consideration of each scenario.

Inter-farm variability in treatment effectiveness may also be associated with differences in the proportion of cattle with cystic ovarian follicles, in diestrus or that were anovular at the time of treatment. The MOFT protocol incorporated additional progesterone and gonadotrophin-releasing hormone treatments, which are more effective for the treatment of anovular disease and cystic ovarian follicles than d-cloprostenol alone [27,43]. The effectiveness of PGOD treatment may be also reduced for treatment of cattle less than 35 days postpartum [44]; although, this represents a minority of cattle in this study, where mean CBSI was 71 (SD 22) days. In contrast, differences between ovulatory responses in cows without a detected estrus but the presence of a corpus luteum at the onset of treatment are equivocal [40,41]. In this study, enrolment was based upon the retention of tail paint for an observation period of 28 days only and determination of luteal status was not performed. Nevertheless, economic comparison of different diagnostic approaches within herds in New Zealand found the most profitable management approach involved treatment with a similar modified Ovsynch protocol, without prior identification of luteal status [4].

Although more complex economic prediction models exist [4,45], partial budget analysis remains a useful and accessible tool for the comparison of changes such as the implementation of novel treatment programs within a farm system [24]. The utility of any model is dependent upon the accuracy of the information entered [16] and to be optimized, farm-specific values are required [25]. The input values used in this study, wherever possible, were based upon primary research or national sales data, although in some circumstances estimates were required. For example, mean 305-day milk yield was used for estimation of additional daily milk yield. It has been suggested that in spring-calving dairy herds additional daily milk production as a result of a reduced calving-conception interval is equivalent to daily peak lactation production [46]. However, this effect may be related to differences in pasture quality and intakes, which may not be relevant in herds housed during calving in autumn. Consequently, mean 305-day milk yield was considered to be a more realistic proxy for estimation of additional daily milk yield. Subsequent sensitivity analysis allowed evaluation of immediate uncertainties associated with input variables to be considered [47]. In circumstances where long-term simulation of an intervention, or the effect of changes in reproductive management on the entire enterprise are required, Markov chain simulation and total enterprise budgeting approaches may be required.

## 5. Conclusions

The relative reproductive and economic performance of a modified Ovsynch protocol, in comparison to a single prostaglandin treatment at mating start date, for treatment of cows without an observed estrus during the 28-days prior to breeding, was evaluated. A modified Ovsynch protocol improved the odds of conception at the beginning of, and throughout, an 84-day breeding season. The effect of modified Ovsynch treatment on barren rate was the key model input associated with profitability. In situations where modified Ovsynch treatment was not associated with improvements in barren rate, profitability was associated with the amount of additional milk and the proportion of beef calves produced. A modified Ovsynch protocol may be an economically viable alternative to prostaglandin treatment of cows without an observed estrus prior to breeding, dependent upon protocol performance and farm dry-off strategy.

## Figures and Tables

**Table 1:** Herd, production and fertility information of the enrolled farms.

Farm		1	2	3
Herd	Herd size	754	378	878
	Breed(s)	HF	HF	HF
Production	Average 305-day milk yield/kg	7564	7189	7396
Reproduction	Conception rate to first AI	61%	61%	39%
	Conception rate	54%	63%	49%
	21 d submission rate	83%	84%	78%
	42 d in-calf rate	61%	80%	51%
	Barren rate	- *	6%	- *

HF, Holstein-Friesian; AI, Artificial insemination. * These herds operated autumn and spring calving systems and accurate determination of barren rate in the autumn group was not possible.

**Table 2:** Key input variables identified during sensitivity analysis. Variation between the low and high figures of these variables resulted in >1% change in the net benefit value.

Input Variable	Low	Expected	High
Reduction in barren rate ^µ^ (%)	2	6	10
Cost of a replacement heifer * (£)	1136	1495	1854
Additional milk produced (Per day, in liters)	15	24	33
Extra beef calves produced within 84 days (%)	−9	−3	+3
Milk price ^γ^ (£, per liter)	0.20	0.28	0.36
Value of a cull cow ^†^ (£)	534	631	728
Additional cost of a progesterone protocol (£, per cow treated)	12	18	24

^µ^ Expected value calculated from Kaplan–Meier survival analysis. * Cost of rearing a replacement heifer in an autumn calving system from birth until first calving [37]. ^†^ Cull cow value estimated from UK cull cow prices sold through auction markets within Great Britain during October 2020, based upon weights of 550 kg, 650 kg, and 750 kg [36]. ^γ^ Milk prices estimated from UK farmgate milk prices during September 2020.

**Table 3:** Summary information of 190 cows recruited to receive either a modified Ovsynch or a prostaglandin based protocol at the mating start date.

Variable	Farm 1	Farm 2	Farm 3	Total
Number of cows enrolled	94	27	69	190
Protocol				
PGOD	42	14	34	90
MOFT	52	13	35	100
Parity				
=1	28 (30%)	9 (33%)	27 (39%)	64 (34%)
=2	17 (18%)	2 (7%)	6 (9%)	25 (13%)
≥3	49 (52%)	16 (60%)	36 (52%)	101 (53%)
BCS ^α^				
≤2.5	10 (13%)	5 (21%)	13 (19%)	28 (17%)
≥2.75	84 (87%)	22 (79%)	56 (81%)	162 (83%)
CBSI (days) Mean (sd)	71 (24)	75 (17)	70 (22)	71 (22)
Calving-first service interval (days) Mean (sd)	79 (25)	73 (19)	81 (25)	79 (25)
Calving-conception interval (days) Mean (sd)	88 (23)	87 (22)	102 (28)	92 (26)
Mating start date-conception interval (days) Median (range)	8 (0–37)	8 (1–49)	29 (0–72)	8 (0–72)
Conception to first AI Number of cows (%)	56 (61%)	21 (78%)	26 (38%)	103 (55%)
21d submission rate Number of cows (%)	87 (93%)	26 (96%)	59 (86%)	174 (92%)
21 day in-calf rate Number of cows (%)	52 (55%)	21 (78%)	22 (32%)	95 (50%)
42 day in-calf rate Number of cows (%)	74 (79%)	25 (93%)	36 (52%)	135 (71%)
84 day in-calf rate Number of cows (%)	75 (80%)	27 (100%)	56 (81%)	158 (83%)

AI, Artificial insemination; BCS, Body condition score; CBSI, Calving breeding-start interval. ^α^ Measured at day 0 during enrolment.

## Data Availability

The data presented in this study are available on request from the corresponding author.

## Supplementary Materials

The following are available online at https://www.mdpi.com/article/10.3390/ani11041031/s1, Table S1: Input values for partial budget and sensitivity analysis.
